# Total Hip Arthroplasty in Post-Bariatric Surgery Patients: Increased Risks and Economic Burden?

**DOI:** 10.3390/healthcare13080887

**Published:** 2025-04-12

**Authors:** Yaron Berkovich, Lahav Rosenberg, Linor Fournier, Yaniv Steinfeld, David Maman

**Affiliations:** 1 Orthopedic Department, Carmel Medical Center, Haifa 3436212, Israel; yaron.berkovich@gmail.com (Y.B.); linorfournier@gmail.com (L.F.); yanivsteinfeld@gmail.com (Y.S.); 2 The Ruth and Bruce Rappaport Faculty of Medicine, Technion Israel Institute of Technology, Haifa 2611001, Israel; 3 Faculty of Medicine, Carol Davila University of Medicine and Pharmacy Bucharest, 050474 Bucharest, Romania; lahavroz@gmail.com

**Keywords:** total hip arthroplasty, bariatric surgery, obesity, surgical outcomes, complications

## Abstract

Background: THA is a widely performed surgical procedure that improves mobility and quality of life in patients with hip joint diseases. The increasing prevalence of obesity has led to a rise in the number of patients undergoing THA following bariatric surgery. This study investigates trends in THA among patients with a history of bariatric surgery, comparing demographics, hospitalization metrics, post-operative complications, and overall surgical outcomes to those without such history. Methods: Using the NIS database (2016–2019), we analyzed a cohort of 1,496,809 THA patients, including 20,429 with a history of bariatric surgery. Propensity score matching was employed to control for confounding factors, resulting in a matched cohort of 20,429 patients in each group. Statistical analyses compared demographic characteristics, comorbidities, hospitalization outcomes, and post-operative complications, with a significance threshold of *p* < 0.05. Results: The proportion of THA patients with prior bariatric surgery increased significantly between 2016 and 2019 (*p* < 0.01). Compared to those without a history of bariatric surgery, these patients were younger (60.3 vs. 66.0 years, *p* < 0.01) and predominantly female (75.0% vs. 55.5%, *p* < 0.01). After PSM, patients with a history of bariatric surgery had a shorter hospital stay (2.17 vs. 2.37 days, *p* = 0.027) but incurred higher hospital charges ($63,631 vs. $62,883, *p* < 0.01). Post-operative complications were significantly higher in this group, with increased risks of hip dislocation (RR = 4.0, 95% CI: 3.4–4.8, *p* < 0.01), surgical site infection (RR = 2.0, 95% CI: 1.8–2.4, *p* < 0.01), pneumonia (RR = 2.5, 95% CI: 2.1–2.8, *p* < 0.01), and intraoperative fracture (RR = 1.6, 95% CI: 1.3–2.0, *p* < 0.01). Conclusions: The rising prevalence of THA in post-bariatric surgery patients highlights the need for optimized perioperative care. Despite shorter hospital stays, these patients face higher complication risks, requiring tailored management. Further research should explore alternative weight management strategies to improve outcomes.

## 1. Introduction

Total hip arthroplasty (THA) is a widely performed and highly effective surgical intervention that significantly improves the quality of life for individuals with hip joint conditions such as osteoarthritis and avascular necrosis. Over the past decade, the global incidence of THA has risen sharply, with a notable increase among younger patients seeking to return to active, physically demanding lifestyles [1].

In parallel, the global prevalence of obesity—defined as a body mass index (BMI) ≥ 30 kg/m^2^—has escalated, increasing by 27.5% in adults and 47.1% in children over the past 30 years [2]. Beyond its well-known systemic effects on cardiovascular and renal health [3], obesity also negatively impacts the musculoskeletal system and is a recognized risk factor for osteoarthritis through both biomechanical and biochemical pathways [4].

As a result, a growing number of obese patients undergo bariatric surgery prior to THA in an effort to reduce perioperative risk and improve long-term surgical outcomes [5,6,7]. This practice is supported by evidence suggesting that significant weight loss prior to joint replacement may reduce complications, enhance recovery, and optimize functional results [8,9,10,11,12,13,14]. Additionally, alternative non-surgical strategies such as medically supervised meal replacement and Mediterranean dietary patterns are being explored for preoperative weight optimization, though their comparative effectiveness remains under investigation.

The primary aim of this study is to compare postoperative outcomes—including complication rates, hospitalization metrics, and patient characteristics—between THA patients with and without a history of bariatric surgery. Leveraging data from the Nationwide Inpatient Sample (NIS), this investigation seeks to provide evidence-based insights into how prior bariatric surgery may influence surgical risks and resource utilization in the context of hip arthroplasty.

A better understanding of this relationship has the potential to improve preoperative risk stratification and guide perioperative care strategies for a growing patient population at the intersection of obesity and joint disease.

### Research Questions

Does a history of bariatric surgery influence complication rates, length of stay, and overall surgical outcomes in patients undergoing total hip arthroplasty?

## 2. Methods

### 2.1. Dataset Description

This analysis utilized data obtained from the Nationwide Inpatient Sample (NIS), which represents the largest publicly accessible inpatient healthcare database in the United States. The cohort comprised 1,496,809 individuals, including 1,474,380 patients who underwent THA without prior bariatric surgery and 20,429 patients who had previously undergone bariatric procedures before their THA.

### 2.2. Study Timeline and Data Source

The study encompassed hospitalizations occurring between 1 January 2016 and 31 December 2019—the most recent four-year span of data available from the NIS at the time of analysis. As a component of the Healthcare Cost and Utilization Project (HCUP), the NIS captures a stratified 20% sample of inpatient discharges from HCUP-participating hospitals, accounting for over seven million unweighted cases annually.

### 2.3. Inclusion Criteria and Exclusion Parameters

Patients who underwent THA were identified through ICD-10 procedure codes, detailed in the Appendix A. Exclusion criteria involved non-elective hospitalizations, revision arthroplasties, and procedures initiated prior to admission.

### 2.4. Statistical Approach and Matching Protocol

All statistical evaluations were conducted using SPSS version 26 and MATLAB 2024. Differences between groups (with and without a bariatric surgery history) were analyzed using independent samples t-tests and cross-tabulations. Risk ratios (RRs) with 95% confidence intervals were calculated using matched cohort proportions to compare complication rates. A *p*-value less than 0.05 denoted statistical significance. To address potential confounding factors, we employed propensity score matching (PSM) via MATLAB, achieving 1:1 matching between groups (*n* = 20,429 in each). Matching variables included demographics, comorbidities (e.g., diabetes, sleep apnea, and hypertension), hospital characteristics (e.g., size and region), urban/rural designation, and median household income. Standardized mean differences (SMDs) were also calculated to assess covariate balance and were all below 0.1, indicating adequate matching.

### 2.5. Identification of Comorbidities and Clinical Outcomes

Comorbidities were identified using ICD-10 codes. Outcomes such as in-hospital mortality, length of stay, total charges, and complications were evaluated only during the index hospitalization, as the NIS does not include post-discharge data. Complications included blood transfusion, surgical site infection, urinary tract infection (UTI), pneumonia, hip dislocation, pulmonary edema, acute kidney injury (AKI), deep vein thrombosis (DVT), intraoperative fracture, and postoperative anemia due to blood loss.

### 2.6. Ethical Considerations

As this study relied exclusively on publicly available, de-identified administrative data, it was exempt from institutional review board (IRB) approval. The nature of the dataset also rendered informed consent unnecessary, since no patient-specific identifiers were present.

## 3. Results

**Figure 1:**
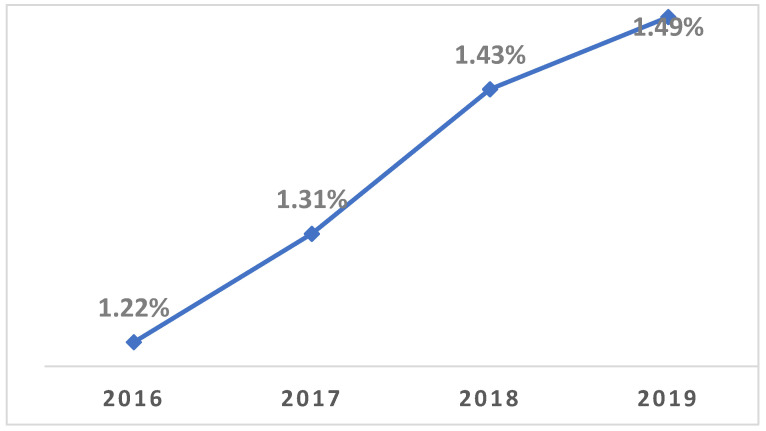
Annual Percentage of patients with history of Bariatric Surgery from all THA Procedures (2016–2019).

There has been a notable trend in the increasing percentage of THA patients with a history of bariatric surgery. As shown in Figure 1, the data indicates a significant upward trend in the proportion of THA patients with a history of bariatric surgery, with a *p*-value less then 0.01.

### 3.1. Comparative Analysis of THA Patients with and Without a History of Bariatric Surgery

Table 1 presents a comparative analysis of 1,474,380 patients undergoing THA without a history of bariatric surgery versus 20,429 patients with a history of bariatric surgery. It highlights the distribution of surgeries, demographic characteristics, and primary expected payers for both groups. The average age of patients undergoing THA without a history of bariatric surgery is significantly higher at 66.0 years compared to 60.3 years for those with a history of bariatric surgery (*p* < 0.01). The proportion of female patients is substantially greater in the group with a history of bariatric surgery, at 75.0% compared to 55.5% in the group without (*p* < 0.01). Primary expected payer categories show notable differences: 55.2% of THA patients without a history of bariatric surgery are covered by Medicare, compared to 43.5% of those with a history of bariatric surgery (*p* < 0.01). Conversely, a larger proportion of patients with a history of bariatric surgery are covered by private insurance, including HMO, at 47.6% compared to 37.2% for those without a history of bariatric surgery (*p* < 0.01). Additionally, patients with a history of bariatric surgery have a slightly higher proportion, covered by Medicaid at 5.6% compared to 4.5% in those without.

### 3.2. Comparative Analysis of Comorbidities in THA Patients with and Without a History of Bariatric Surgery

Table 2 provides a comparative analysis of comorbidities in patients undergoing THA with and without a history of bariatric surgery. Patients with a history of bariatric surgery exhibit significantly higher rates of obstructive sleep apnea (22.1% vs. 9.9%, *p* < 0.01), chronic anemia (8.4% vs. 5.6%, *p* < 0.01), osteoporosis (5.8% vs. 4.4%, *p* < 0.01), and obesity (54.2% vs. 23.3%, *p* < 0.01). They also have a higher prevalence of chronic use of anticoagulants (7.6% vs. 5.5%, *p* < 0.01) and diabetes mellitus (21.2% vs. 14.9%, *p* < 0.01).

Patients without a history of bariatric surgery demonstrate higher rates of dyslipidemia (43% vs. 34%, *p* < 0.01), chronic kidney disease (6.3% vs. 5.4%, *p* < 0.01), and chronic lung disease (6.3% vs. 5%, *p* < 0.01). Additionally, this group has a higher prevalence of hypertension (52.5% vs. 55.3%, *p* < 0.01) and alcohol abuse (1.3% vs. 1.5%, *p* < 0.01), while the difference in congestive heart failure (1.1% vs. 1.5%, *p* = 0.698) was not statistically significant.

### 3.3. Propensity Score-Matched Analysis of Comorbidities in THA Patients with and Without a History of Bariatric Surgery

In order to address potential selection bias and baseline differences in comorbidities, a propensity score-matched analysis was performed, ensuring that the two groups compared were statistically equivalent, thereby minimizing selection bias. Propensity score-matched analysis is a statistical technique used in observational studies to create comparable groups by matching individuals based on their likelihood of being in either group. This approach helps balance participant characteristics, reduces the impact of confounding variables, and improves the reliability of conclusions. It aims to replicate the random assignment process seen in experimental studies.

Table 3 provides a detailed comparison of comorbidities between patients undergoing THA with and without a history of bariatric surgery after propensity score matching. This analysis includes 20,429 patients in each group, ensuring a balanced comparison. The results reveal no statistically significant differences across a range of demographic and comorbidity parameters, including age, gender, primary expected payer, and most comorbid conditions. These findings underscore the effectiveness of the propensity score-matching process in reducing selection bias and allowing for a fair comparison between the two groups.

### 3.4. Comparison of Hospitalization Outcomes in Propensity Score-Matched THA Patients

Hospitalization outcomes were assessed in the propensity score-matched cohorts of total hip arthroplasty (THA) patients with and without a history of bariatric surgery. Table 4 summarizes the mean length of stay and total charges for both groups.

The results reveal significant differences in these outcomes. Patients with a history of bariatric surgery had a shorter average length of stay (2.17 days, Std. deviation 2.1) compared to those without a history of bariatric surgery (2.37 days, Std. deviation 1.6, *p* = 0.027). Conversely, the total hospital charges were slightly higher for patients with a history of bariatric surgery ($63,631, Std. deviation 36,975) compared to those without a history of bariatric surgery ($62,883, Std. deviation 33,835, *p* < 0.01).

### 3.5. Comparison of Select Postoperative Complications in Propensity Score-Matched THA Patients with and Without a History of Bariatric Surgery

Table 5 presents a comparison of select complications in propensity score-matched cohorts of THA patients with and without a history of bariatric surgery. The findings reveal that, after matching, patients with a history of bariatric surgery did not exhibit significantly worse outcomes for DVT and blood loss anemia. Moreover, the rate of AKI was slightly lower in the bariatric surgery group (1.3% vs. 1.6%, *p* = 0.014).

The in-hospital mortality rate was <0.1% and did not significantly differ between the groups. Pulmonary edema was rare and also showed no statistically significant difference.

### 3.6. Elevated Risk Ratios for Complications in THA Patients with a History of Bariatric Surgery Compared to Propensity Score-Matched Patients Without Such History

**Figure 2:**
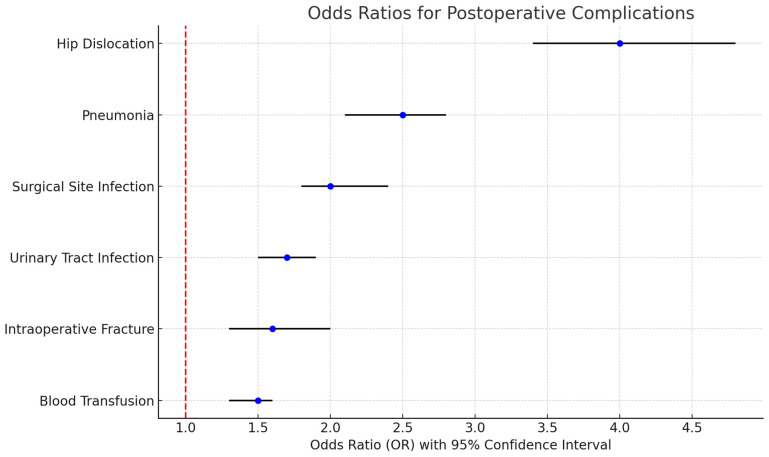
Risk ratios for complications in THA patients with a history of bariatric surgery compared to those without a history of bariatric surgery in a propensity score-matched cohort.

Figure 2 illustrates the risk ratios (RR) for various complications in THA patients with a history of bariatric surgery compared to propensity score-matched patients without a history of bariatric surgery.

The findings indicate statistically significant elevated risks across all complications.

For blood transfusion, the risk ratio is 1.5, with a 95% confidence interval (CI) of 1.3 to 1.6 (*p* < 0.01). Intraoperative fractures show a risk ratio of 1.6, with a 95% CI of 1.3 to 2.0 (*p* < 0.01). The risk ratio for urinary tract infection is 1.7, with a 95% CI of 1.5 to 1.9 (*p* < 0.01). Surgical site infections demonstrate a risk ratio of 2.0, with a 95% CI of 1.8 to 2.4 (*p* < 0.01). Pneumonia exhibits a higher risk ratio of 2.5, with a 95% CI of 2.1 to 2.8 (*p* < 0.01). The highest observed risk is for hip dislocation, with a risk ratio of 4.0 and a 95% CI of 3.4 to 4.8 (*p* < 0.01). Blood transfusion occurred in 3.1% of patients in the control group and 4.5% in the post-bariatric surgery group. Intraoperative fracture occurred in 0.4% of patients in the control group and 0.6% in the post-bariatric surgery group. Urinary tract infections occurred in 2.5% of patients in the control group and 4.2% in the post-bariatric surgery group. Surgical site infections occurred in 0.8% of patients in the control group and 1.6% in the post-bariatric surgery group. Pneumonia occurred in 1.0% of patients in the control group and 2.5% in the post-bariatric surgery group. Hip dislocations occurred in 0.3% of patients in the control group and 1.2% in the post-bariatric surgery group.

## 4. Discussion

### 4.1. Main Findings

This study reveals a significant increase in THA procedures among patients with a history of bariatric surgery between 2016 and 2019. Despite preoperative weight loss, these patients face elevated risks for specific postoperative complications. Additionally, while their hospital stays are slightly shorter, the associated healthcare costs are marginally higher compared to patients without prior bariatric surgery.

#### 4.1.1. Increased Prevalence of THA in Post-Bariatric Surgery Patients

The rising prevalence of THA in post-bariatric surgery patients may be attributed to the global increase in obesity and the subsequent adoption of bariatric procedures as weight-loss interventions. Bariatric surgery is often recommended before THA to mitigate obesity-related surgical risks. Studies have shown that obese patients undergoing bariatric surgery prior to THA experience reduced medical complications and lower revision rates over time [15,16,17]. However, the benefits of preoperative weight loss on surgical outcomes remains a topic of debate, with some studies suggesting limited impact on perioperative complications.

#### 4.1.2. Economic Impact and Length of Hospital Stay

Our analysis indicates that patients with a history of bariatric surgery have a marginally shorter hospital stay following THA (2.17 vs. 2.37 days). This finding aligns with research suggesting that preoperative weight loss can lead to a reduced length of stay [12,18]. Although the difference in LOS (2.17 vs. 2.37 days) reached statistical significance (*p* = 0.027), the clinical relevance of a 0.2-day reduction is limited. Despite the shorter hospitalization, these patients incur slightly higher total hospital charges. This paradox may be due to the need for specialized perioperative care or management of comorbidities, which can increase overall healthcare costs.

#### 4.1.3. Increased Postoperative Complications

Post-bariatric surgery patients undergoing THA face elevated risks for certain complications. Our study found higher incidences of surgical site infections, pneumonia, urinary tract infections, and hip dislocations in this group. These findings are consistent with literature indicating that while bariatric surgery may reduce some medical complications, it does not eliminate the heightened risk of surgical complications. The metabolic changes following bariatric surgery, particularly the potential for nutritional deficiencies and altered bone metabolism, may contribute to complications such as fractures, infections, and delayed healing [19,20,21,22,23]. Key deficiencies such as vitamin D, calcium, and protein malnutrition are common following bariatric procedures and may compromise bone health, immune function, and tissue healing. These deficiencies likely contribute to the observed increase in fracture and infection rates, highlighting the need for careful perioperative nutritional assessment and supplementation in this patient population.

RRs were presented as they better represent the relative likelihood of complications within the dataset’s limitation of capturing only in-hospital outcomes. Raw incidence rates may underestimate events that occur post-discharge (e.g., dislocation), so RRs are more reflective of relative risk in this context.

#### 4.1.4. Short-Term Benefits vs. Long-Term Outcomes

While preoperative bariatric surgery may offer short-term benefits, such as reduced lengths of stay and certain medical complications, the long-term advantages are less clear. Some studies suggest that significant preoperative weight loss does not necessarily correlate with improved long-term functional outcomes after THA [20,24,25]. However, weight reduction could potentially enhance the ability of patients to participate in postoperative physiotherapy, potentially leading to better functional recovery over time [26]. Further research is needed to elucidate the long-term impact of bariatric surgery on THA outcomes.

#### 4.1.5. Emerging Pharmacological Interventions

Recent advancements in pharmacotherapy, particularly the use of glucagon-like peptide-1 receptor agonists (GLP-1 RAs) such as semaglutide, have shown promise in facilitating weight loss among obese patients. Studies indicate that the perioperative use of GLP-1 RAs in morbidly obese patients is associated with reduced risks of acute periprosthetic joint infection and 90-day hospital readmission rates [27,28]. These findings suggest that GLP-1 RAs could serve as an alternative to bariatric surgery for weight management prior to THA, potentially mitigating surgical risks associated with obesity. However, these observations remain speculative, and further randomized controlled trials are necessary to determine the true effect and mechanism of action.

### 4.2. Limitations and Future Directions

This study has several limitations. The reliance on administrative data may introduce coding inaccuracies and lacks detailed clinical information [29,30,31], such as BMI, medication use, anesthesia type, or specific surgical indications (e.g., OA vs. AVN). The timing of bariatric surgery relative to THA could not be assessed. In-hospital data only captures complications occurring during admission, not 30- or 90-day events. Despite propensity score matching, residual confoundment is possible. Alternative weight-loss strategies, such as structured dietary programs or supervised meal replacement, were not assessed but merit future comparison. Future research should also explore the efficacy of GLP-1 RAs and other pharmacologic weight-loss agents compared to bariatric surgery. Prospective studies with detailed, longitudinal data are warranted to inform perioperative care and optimize outcomes.

## 5. Conclusions

The rising prevalence of THA in post-bariatric surgery patients highlights the need for optimized perioperative care. Despite shorter hospital stays, these patients face higher complication risks, requiring tailored management. Further research should explore alternative weight management strategies to improve outcomes.

## Figures and Tables

**Table 1:** Demographic and Payer Characteristics of THA patients with and without a history of bariatric surgery.

Parameter	No History of Bariatric Surgery	History of Bariatric Surgery	Significance
Total Surgeries	1,474,380	20,429	-
Average Age (y)	66.0	60.3	*p* < 0.01
Female (%)	55.5	75.0	*p* < 0.01
payer—Medicare (%)	55.2	43.5	*p* < 0.01
payer—Medicaid (%)	4.5	5.6	
payer—private including HMO (%)	37.2	47.6	
payer—self-pay (%)	0.7	0.3	
payer—no charge (%)	0	0	
payer—other (%)	2.3	2.9	

**Table 2:** Prevalence of comorbidities in THA patients with and without a history of bariatric surgery.

Parameter	No History of Bariatric Surgery	History of Bariatric Surgery	Significance
Hypertension (%)	52.5	55.3	*p* < 0.01
Dyslipidemia (%)	43	34	*p* < 0.01
Obstructive Sleep Apnea (%)	9.9	22.1	*p* < 0.01
Chronic Anemia (%)	5.6	8.4	*p* < 0.01
Alcohol Abuse (%)	1.3	1.5	*p* < 0.01
Osteoporosis (%)	4.4	5.8	*p* < 0.01
Chronic Kidney Disease (%)	6.3	5.4	*p* < 0.01
Congestive Heart Failure (%)	1.1	1.5	*p* < 0.01
Chronic Lung Disease (%)	6.3	5	*p* < 0.01
Diabetes Mellitus (%)	14.9	21.2	*p* < 0.01
Obesity (%)	23.3	54.2	*p* < 0.01
Chronic use of anticoagulants (%)	5.5	7.6	*p* < 0.01

**Table 3:** Comparison of demographic and clinical data in propensity score-matched cohorts of THA patients with and without a history of bariatric surgery.

Parameter	No History of Bariatric Surgery	History of Bariatric Surgery	Sign.
Total Surgeries	20,429	20,429	-
Average Age (y)	60.2	60.3	*p* = 0.13
Female (%)	74.8	75	*p* = 0.61
payer—Medicare (%)	42.7	42.7	*p* = 0.32
payer—Medicaid (%)	5.8	6.1	
payer—private including HMO (%)	48.4	47.9	
payer—self-pay (%)	0.5	0.5	
payer—no charge (%)	0.1	0	
payer—other (%)	2.5	2.8	
Hypertension (%)	55.7	55.3	*p* = 0.40
Dyslipidemia (%)	34	34	*p* = 1
Obstructive Sleep Apnea (%)	21.9	22.1	*p* = 0.64
Chronic Anemia (%)	8.1	8.4	*p* = 0.10
Alcohol Abuse (%)	1.6	1.5	*p* = 0.16
Osteoporosis (%)	5.5	5.8	*p* = 0.54
Chronic Kidney Disease (%)	5	5.4	*p* = 0.06
Congestive Heart Failure (%)	1.2	1.5	*p* = 0.09
Chronic Lung Disease (%)	5.1	5.0	*p* = 0.82
Diabetes Mellitus (%)	21	21.2	*p* = 0.63
Obesity (%)	54.6	54.2	*p* = 0.40
Chronic use of anticoagulants (%)	7.8	7.6	*p* = 0.34

**Table 4:** Comparison of hospitalization outcomes in propensity score-matched cohorts of THA patients with and without a history of bariatric surgery.

	No History of Bariatric Surgery	History of Bariatric Surgery	Significance
Length of stay mean in days	2.37 (Std. deviation 1.6)	2.17 (Std. deviation 2.1)	*p* = 0.03
Total charges mean in $	62,883 (Std. deviation 33,835)	63,631 ( Std. deviation 36,975)	*p* < 0.01

**Table 5:** Comparison of select postoperative complications in propensity score-matched THA patients with and without a history of bariatric surgery.

Parameter	No History of Bariatric Surgery	History of Bariatric Surgery	Significance
AKI (%)	1.6	1.3	*p* = 0.01
DVT (%)	0.1	0.1	*p* = 0.16
Blood Loss Anemia (%)	20.1	20.5	*p* = 0.36

## Data Availability

The original contributions presented in the study are included in the article; further inquiries can be directed to the corresponding author.

## Abbreviations

CKD	Chronic Kidney Disease
DM	Diabetes Mellitus
ICD-10	International Classification of Diseases, 10th Revision
LOS	Length of Stay
NIS	Nationwide Inpatient Sample
OR	Odds Ratio
OSA	Obstructive Sleep Apnea
SSI	Surgical Site Infection
THA	Total Hip Arthroplasty
UTI	Urinary Tract Infection

## Appendix A

**ICD 10 CODES/PROCEDURE CODE.**	
I5021, I5031, I5033, I5041, I5043	Heart Failure
N170, N171, N172, N178, N179	Acute Kidney Injury
I2101, I2102, I2109, I211, I2119, I2111, I212, I2129, I213, I214, I219	Acute Coronary Artery Disease
I60, I61, I62, I63, I650, I688, O873, O2250, O2251, O2252	Stroke
J810, J811, I501	Pulmonary Edema
I10 (start with)	Hypertension
D62 (start with)	Blood Loss Anemia
J189, J159, J22	Pneumonia
I2602, I2609, I2692, I2699	Pulmonary Embolism
I82401, I82402, I82403, I82409, I82411, I82412, I82413, I82419, I82421, I82422, I82423, I82429	DVT
E78 (start with)	Dyslipidemia
G473	Obstructive Sleep Apnea
D64 (start with)	Chronic Anemia
F10	Alcohol Abuse History
M81, M82	Osteoporosis
F (start with)	Mental Disorders
G20 (start with)	Parkinson Disease
E11 (start with)	Type 2 Diabetes Mellitus
N18 (start with)	Chronic Kidney Disease
I500, I501, I509	Congestive Heart Failure
J44 (start with)	Chronic Lung Disease
