# Correction: Berkovich et al. Total Hip Arthroplasty in Post-Bariatric Surgery Patients: Increased Risks and Economic Burden? *Healthcare* 2025, *13*, 887

**DOI:** 10.3390/healthcare13182305

**Published:** 2025-09-15

**Authors:** Yaron Berkovich, Lahav Rosenberg, Linor Fournier, Yaniv Steinfeld, David Maman

**Affiliations:** 1 Orthopedic Department, Carmel Medical Center, Haifa 3436212, Israel; yaron.berkovich@gmail.com (Y.B.); linorfournier@gmail.com (L.F.); yanivsteinfeld@gmail.com (Y.S.); 2 The Ruth and Bruce Rappaport Faculty of Medicine, Technion Israel Institute of Technology, Haifa 2611001, Israel; 3 Faculty of Medicine, Carol Davila University of Medicine and Pharmacy Bucharest, 050474 Bucharest, Romania; lahavroz@gmail.com

There was an error in the original publication [1]. The Appendix section is missing.

A correction has been made in that we added an appendix file containing the ICD-10 codes used in the study to enhance the transparency of the methodology.

## Appendix A

**ICD 10 CODES/PROCEDURE CODE.**	
I5021, I5031, I5033, I5041, I5043	Heart Failure
N170, N171, N172, N178, N179	Acute Kidney Injury
I2101, I2102, I2109, I211, I2119, I2111, I212, I2129, I213, I214, I219	Acute Coronary Artery Disease
I60, I61, I62, I63, I650, I688, O873, O2250, O2251, O2252	Stroke
J810, J811, I501	Pulmonary Edema
I10 (start with)	Hypertension
D62 (start with)	Blood Loss Anemia
J189, J159, J22	Pneumonia
I2602, I2609, I2692, I2699	Pulmonary Embolism
I82401, I82402, I82403, I82409, I82411, I82412, I82413, I82419, I82421, I82422, I82423, I82429	DVT
E78 (start with)	Dyslipidemia
G473	Obstructive Sleep Apnea
D64 (start with)	Chronic Anemia
F10	Alcohol Abuse History
M81, M82	Osteoporosis
F (start with)	Mental Disorders
G20 (start with)	Parkinson Disease
E11 (start with)	Type 2 Diabetes Mellitus
N18 (start with)	Chronic Kidney Disease
I500, I501, I509	Congestive Heart Failure
J44 (start with)	Chronic Lung Disease

The authors state that the scientific conclusions are unaffected. This correction was approved by the Academic Editor. The original publication has also been updated.
